# Disability trajectories and mortality in older adults with different cognitive and physical profiles

**DOI:** 10.1007/s40520-019-01297-1

**Published:** 2019-08-30

**Authors:** Giulia Grande, Davide L. Vetrano, Laura Fratiglioni, Anna Marseglia, Nicola Vanacore, Erika Jonsson Laukka, Anna-Karin Welmer, Debora Rizzuto

**Affiliations:** 1 Aging Research Center, Department of Neurobiology, Care Sciences and Society, Karolinska Institutet and Stockholm University, Stockholm, Sweden; 2 Stockholm Gerontology Research Centre, Stockholm, Sweden; 3 Department of Geriatrics, Catholic University of Rome, Rome, Italy; 4 Centro di Medicina dell’Invecchiamento, Fondazione Policlinico A. Gemelli, Rome, Italy; 5 National Centre for Disease Prevention and Health Promotion, National Institute of Health, Rome, Italy

**Keywords:** Cognitive impairment, Walking speed, Survival, Disability, Population-based study

## Abstract

**Background:**

Cognitive and physical deficits independently raise the risk for negative events in older adults. Less is known about whether their co-occurrence constitutes a distinct risk profile. This study quantifies the association between cognitive impairment, no dementia (CIND), slow walking speed (WS) and their combination and disability and mortality.

**Methods:**

We examined 2546 dementia-free people aged ≥ 60 years, part of the Swedish National study on Aging and Care in Kungsholmen (SNAC-K) up to 12 years. The following four profiles were created: (1) healthy profile; (2) isolated CIND (scoring 1.5 SD below age-specific means on at least one cognitive domain); (3) isolated slow WS (< 0.8 m/s); (4) CIND+ slow WS. Disability was defined as the sum of impaired activities of daily living and trajectories of disability were derived from mixed-effect linear regression models. Piecewise proportional hazard models were used to estimate mortality rate [hazard ratios (HRs)]. Population attributable risks of death were calculated.

**Results:**

Participants with both CIND and slow WS had the worst prognosis, especially in the short-term period. They experienced the steepest increase in disability and five times the mortality rate (HR 5.1; 95% CI 3.5–7.4) of participants free from these conditions. Similar but attenuated results were observed for longer follow-ups. Co-occurring CIND and slow WS accounted for 30% of short-term deaths.

**Conclusions:**

Co-occurring cognitive and physical limitations constitute a distinct risk profile in older people, and account for a large proportion of short-term deaths. Assessing cognitive and physical function could enable early identification of people at high risk for adverse events.

**Electronic supplementary material:**

The online version of this article (10.1007/s40520-019-01297-1) contains supplementary material, which is available to authorized users.

## Introduction

Cognitive and physical functioning are crucial determinants of health that decline as part of the aging process [1]. An impairment in these functions independently leads to higher morbidity burden, excess dependence, and ultimately, shorter survival [2, 3]. Although they have most frequently been studied separately, physical and cognitive impairments often co-occur in later life [4] and tend to interact and influence each other, leading to complex health profiles [5]. It is thus plausible to hypothesize that their co-occurrence may enhance the development of negative health events.

Over the last few years, a handful of studies have investigated the simultaneous presence of physical and cognitive impairments and their impact on health status, but results have been conflicting [6–9] and it remains unclear whether—and to what extent—co-occurring cognitive and physical limitations constitute a high-risk profile, distinct from that of impairment in one single domain. With few exceptions [10, 11], the majority of those studies reported an increased risk of adverse outcomes along with the presence of both cognitive and physical impairments [7]. However, only one study has investigated the relationship between cognitive and physical domains and adverse outcomes (i.e., dementia) over a short and long term [9], and none of them have quantified trajectories of functional decline associated with deficits in cognition and physical function.

Physical and cognitive functions are assessed in multiple ways across different studies, further complicating the interpretation of the results [12]. Walking speed, one of many measures of physical function, is a practical and exhaustive proxy for frailty that has been successfully used in clinical and research settings [13, 14]. It is a simple and accessible summary indicator of the vitality of an individual, as it integrates clinical and subclinical disturbances in several organs and systems, many of which may affect dependence and survival [13, 15, 16]. Choosing a measure of cognitive function is even more challenging. Many tests are available, and researchers propose different cutoffs [17]. However, an extensive neuropsychological battery that investigates multiple cognitive domains can detect mild and initial deficits more sensitively than a single score.

Congruent with our hypothesis are also the findings from our and others´ previous studies [5, 18] which show that individuals with both somatic and mental diseases are at highest risk of further deterioration in health, suggesting a close link between the physical and mental systems. As a consequence, it might be important to consider physical and cognitive functions in older adults as related rather than separate phenomena.

In this study, we aim to test this hypothesis by investigating the occurrence of cognitive impairment, slow walking speed, and their combination, and quantifying their association with disability and mortality in older adults over a period of 12 years. Studying the prognosis of these dysfunctions will provide further information to better tailor and implement individualized preventive and therapeutic strategies.

## Materials and methods

### Study population

Data were extracted from the ongoing population-based Swedish National study on Aging and Care in Kungsholmen (SNAC-K), which has been described previously [19]. Briefly, the study population includes people aged 60+ years living either at home or in institutions in the Kungsholmen district of Stockholm. A random sample of 5111 people from 11 age cohorts was selected at baseline (March 2001 through August 2004), and 73.3% (*n* = 3363) of the eligible people were examined. Participants aged < 72 years (younger cohort) were followed up every 6 years, and those aged ≥ 78 (older cohort), every 3 years.

In accordance with the study protocol of SNAC-K, people with a Mini Mental State Examination (MMSE) score < 10 (*n* = 106) and participants with severe vision or hearing problems (*n* = 9) did not undergo the neuropsychological battery. In addition, 390 participants declined to participate in the cognitive tests, and 10 died before the scheduled neuropsychological assessment, leaving a sample of 2848 people. People who were not tested were more likely to be older, women, and affected by dementia (*p* < .001 for all) [20]. We excluded from the analyses of the present study prevalent dementia cases (*n* = 83), people with a diagnosis of schizophrenia (*n* = 11) and intellectual disability/developmental disorder (*n* = 2), and those for whom data on one or more cognitive domains (*n* = 129) or walking speed (*n* = 77) were missing, leaving a study sample of 2546 participants. Figure S1 depicts the flowchart of the study population. Those who had missing data were more likely to be women, older, to have a lower level of education, to live in institution, and to have a greater number of chronic diseases than those in the final study sample (*p* < .001 for all).

The SNAC-K study was approved by the Regional Ethical Review Board in Stockholm, Sweden. Written informed consent was collected from participants, or, for participants with cognitive impairment, from the next of kin. The results of the present study were reported in accordance with the STROBE recommendations.

### Definitions of main variables and covariates

*Data collection* At baseline and each follow-up visit, data were collected at our research center via face-to-face interviews by nurses, clinical examinations and laboratory tests by physicians, and a cognitive battery administered by psychologists. Data collection lasted up to 5 h per participant. For those who agreed to participate but were unable to come to the research center, home visits were conducted.

*Walking speed* (WS) WS was assessed by asking the participants to walk at their usual speed over six or over 2.4 m, if the participant reported walking quite slowly or the evaluation was conducted at the participant’s home and space was restricted [21]. Previous reports have shown that the use of different distances in the assessment of WS is highly comparable [22]. If the participant was unable to walk or attempted unsuccessfully to walk, a value of zero was recorded. WS was reported in meters/second (m/s). A cutoff of < 0.8 m/s was used to define slow walking speed. This cutoff has been widely used to identify a subgroup of people with an increased likelihood of poor health and function [13].

*Cognitive impairment, no dementia* (CIND) CIND, as compared with mild cognitive impairment (MCI), is a broader definition of cognitive impairment that does not require the presence of cognitive complaints and allows the presence of functional disability [23, 24]. The latter characteristic appears preferable when studying the interplay between cognitive and physical function. In addition, the presence of subjective cognitive complaint, part of the operationalization of MCI, which is highly predictive of dementia in people referred to a memory clinic or to general practitioners [25], shows less accuracy in population-based studies because of a higher number of false positive and false negative results [26–29]. Based on example of previous reports [17, 23], we operationalized CIND as the presence of objective cognitive impairment, defined as scoring 1.5 standard deviations (SDs) or more below age group-specific means in at least one domain of the neuropsychological assessment, in the absence of dementia [23]. Moderate to severe CIND was defined as scoring 2.0 SDs or more below the age group-specific means. The extensive neuropsychological battery performed by trained psychologists included seven tests to measure the following cognitive domains: episodic memory (Free recall), executive function (Trail Making Test, part B), language (Category and Letter fluency), visuospatial abilities (Mental rotation) and perceptual speed (Digit cancellation and Pattern comparison). For each test, the raw scores were standardized into *z* scores using the baseline mean and SD. When more than one test was available, the cognitive domain was created by averaging the *z* scores of the domain-specific tests.

*Functional profiles* The following mutually exclusive functional profiles were created by combining CIND and WS: (1) healthy functional profile—participants without CIND and with a WS ≥ 0.8 m/s (reference group), (2) participants with isolated CIND, (3) participants with isolated slow WS, (4) participants with both CIND and slow WS.

*Disability status* Participants’ basic activities of daily living (ADL) and instrumental activities of daily living (IADL) were assessed by trained nurses at baseline and every follow-up through a structured questionnaire primarily to the participant, and, in case of cognitive impairment, to the proxy. ADL assessed dependence in bathing, dressing, toileting, transferring, continence, and feeding. IADL assessed the ability to use the phone, shop, prepare food, participate in housekeeping tasks, do the laundry, use public transportation, take medications, and handle finances. People living in institutions were assumed to depend on others for grocery shopping, meal preparation, housekeeping, and laundry. Since some of the IADL may be not performed as a matter of habit (e.g., men might more rarely report taking care of laundry, housekeeping, etc.), the participant had the option of answering: “I do not, but I could”. The answers to these questions have been taken into account to build the final score. In keeping with a previous report [30], the number of ADL and IADL limitations was summed together, and a disability score (range 0–14) was created. Changes in ADL and IADL scores have been also considered separately.

*Vital status* Information about the vital status of the participants was obtained from the Swedish Cause of Death Register. Data were available up to December 2016.

*Covariates* Data on age, sex, and education were derived from the nurse’s interview at baseline. Socioeconomic position is a variable derived from the longest held occupation and has been categorized into three groups: (1) blue collar workers (e.g., unskilled/skilled goods producing); (2) white collar workers (e.g., junior office worker, less than 3 years education after elementary school); (3) entrepreneurs (e.g., self-employed). Level of physical activity is based on a questionnaire administered to the participants, which assesses both the frequency and the intensity of these activities. Physical inactivity is defined as being physically active for less than once a week in light and/or intensive activity. In SNAC-K, comprehensive clinical evaluations, blood tests, use of drugs, and national inpatient and outpatient registers were used to identify chronic diseases [31]. All the diagnoses were coded in keeping with the International Classification of Diseases, 10th edition (ICD-10). For the present study, we took the following diseases into consideration: cardio- and cerebrovascular diseases, hypertension, chronic obstructive pulmonary disorders, solid neoplasms, depression, and mood disorders. The diagnosis of clinical dementia is made in accordance with the criteria of the DSM-IV-TR and follows a three-step procedure. A first preliminary diagnosis is made by the examining physician, reviewed by a second physician involved in the data collection. In case of disagreement between the two physicians, the final diagnosis is made by a neurologist external to the data collection process. Body mass index (BMI) was obtained by dividing the participants’ weight by their squared height (kg/m^2^). Underweight being a reliable indicator of malnutrition, known to be the downstream event of several concurrent chronic diseases of the older person, and malnutrition being a potential confounder in the studied association (i.e., malnourished people are more likely to suffer from cognitive impairment, slow WS, and have negative outcomes such as disability and shorter survival), we further adjusted our analyses for BMI < 18.5 kg/m^2^, considered a proxy of malnutrition [32].

### Statistical analysis

Trajectories of disability over the 12 years of follow-up were obtained with multilevel mixed-effect linear regression models, adjusting for age, sex, education, chronic diseases, and malnutrition. Follow-up time was modeled through unrestricted cubic splines with four knots (0, 3, 6, and 9 years).

Since the strength of the association of the functional profiles on mortality rate varied upon the timescale, we adopted piecewise proportional hazard models, which account for the non-proportionality of hazards by modeling the hazard ratio (HR) as a step function of the follow-up time, which was divided into intervals. In each interval, the HR is assumed to be constant but is allowed to vary between these intervals [33]. The population attributable risk (PAR) of death was calculated by using the formula for survival studies to estimate the proportion of deaths averted in the hypothetical scenario that it would be possible to eliminate CIND and slow WS [34, 35]. Survival time was defined as the time between the baseline evaluation date and either death or the end of follow-up (December 2016), whichever came first. We tested for interactions between functional profiles and sex and age, and stratified analyses were also performed.

*Sensitivity analyses* To assess the strength and consistency of our results, sensitivity analyses were performed. First, the same association (i.e., between the functional profiles and disability and mortality) was assessed by entering the functional profiles into the models as time-changing variables. This analysis assessed the association between the functional profiles and the outcomes, accounting for the change of the exposure status over time. Second, since the accumulation of disabilities over time could be due to the fact that people with CIND might develop dementia over time, we repeated the analyses excluding incident cases of dementia within the first 6 years of follow-up. Third, we took into account the missing data in the definition of the functional profiles at baseline, repeating the analyses with multiple imputations by chained equation (MICE), obtaining five imputed datasets. The estimates of these datasets were pooled using Rubin’s rule for valid statistical inferences. All the relevant covariates (i.e., age, sex, education, walking speed, chronic diseases, and malnutrition) included in the major analyses were used in the multiple imputation models, as were the outcomes (disability and time to death). Finally, to take informative dropouts (due to death) into account, we used joint modeling to simultaneously model the association between the functional profiles and disability (using linear mixed effects models) and survival (using the Weibull model), adjusting for age, sex, education, chronic diseases, and malnutrition [36].

We repeated all the analyses on disability considering also the changes in ADL and IADL scores separately. Finally, all the analyses were repeated considering only those participants with moderate to severe CIND as cognitively impaired and using a different cutoff for slow WS (i.e., 1 m/s) [13].

A *p* value < 0.05 was considered statistically significant in all the analyses. All analyses were performed using Stata version 14 (StataCorp, Texas, USA).

## Results

### Characteristics of the study population

The mean age of the 2546 participants was 72 years, 61% were women and 37% had a university education. The sample characteristics at baseline by age group and sex are shown in Table 1.

**Table 1:** Sample baseline characteristics by age and sex

	Age (years)			Sex		
	60–78 years *N* = 1582	≥78 years *N* = 964	*p* value	Women *N* = 1561	Men *N* = 985	*p* value
Women	900 (56.9)	661 (68.6)	< .001	–	–	–
Age (mean ± SD)	–	–	–	73.1 ± 10.0	70.6 ± 10.1	< .001
Education						
Elementary school	125 (7.9)	231 (24.0)	< .001	234 (15.0)	122 (12.4)	< .001
High school	722 (45.6)	525 (54.5)		835 (53.5)	412 (41.8)	
University	735 (46.5)	208 (21.5)		492 (31.5)	451 (45.8)	
Socioeconomic position						
Blue collar workers	248 (15.7)	275 (28.6)	< .001	366 (23.5)	157 (16.0)	< .001
White collar works	1160 (73.3)	599 (62.3)		1086 (69.6)	673 (68.3)	
Entrepreneurs	174 (11.0)	88 (9.1)		108 (6.9)	154 (15.7)	
Functional assessment						
Slow walking speed (< 0.8 m/s)	95 (6.0)	397 (41.2)	< .001	356 (22.8)	136 (13.8)	< .001
Walking speed (m/s) (mean ± SD)	1.23 ± 0.30	0.82 ± 0.37	< .001	1.03 ± 0.39	1.15 ± 0.37	.074
Cognitive impairment, no dementia	375 (23.7)	303 (31.4)	< .001	450 (28.8)	228 (23.2)	.002
Physical inactivity	300 (19.0)	327 (33.9)	< .001	231 (23.5)	396 (25.4)	.274
Clinical assessment						
No. of chronic diseases (mean ± SD)	2.9 ± 1.8	4.6 ± 1.8	< .001	3.7 ± 2.0	3.4 ± 1.9	.002
No. of medications (mean ± SD)	2.9 ± 3.0	4.9 ± 3.3	< .001	4.1 ± 3.2	3.0 ± 3.1	< .001
Hypertension	1073 (67.8)	818 (84.6)	< .001	1169 (74.9)	722 (73.4)	.372
Cardio- and cerebrovascular diseases^a^	292 (18.5)	431 (44.7)	< .001	377 (24.2)	346 (35.1)	< .001
Depression and mood disorders	125 (7.9)	80 (8.3)	.721	147 (9.4)	58 (5.9)	.001
Solid neoplasms	113 (7.1)	110 (11.4)	< .001	120 (7.7)	103 (10.5)	.016
Chronic obstructive pulmonary disorders	60 (3.8)	56 (5.8)	.018	67 (4.3)	49 (5.0)	.421
Malnutrition^b^	15 (1.0)	43 (4.5)	< .001	44 (2.8)	14 (1.4)	.021
Disability						
Disability score (range 0–14) (mean ± SD)^c^	0.06±	0.8 ± 1.7	< .001	0.38 ± 1.2	0.23 ± 0.9	.007
Impairment in at least 1 ADL	9 (0.6)	55 (5.7)	< .001	49 (3.1)	15 (1.5)	.011
Impairment in at least 1 IADL	59 (3.8)	234 (25.0)	< .001	202 (13.2)	91 (9.5)	.005

Unless otherwise specified, figures show number (%). *P* values were obtained through Chi squared test for categorical and *t* test for continuous variables

*CI* confidence interval, *SD* standard deviation, *CIND* cognitive impairment, no dementia, *ADL* activities of daily living, *IADL* instrumental activities of daily living

^a^Defined as ischemic heart disease, heart failure, diabetes, atrial fibrillation, and stroke

^b^Malnutrition, defined as body mass index < 18.5 kg/m^2^

^c^Combining the six basic and eight instrumental activities of daily living, analysis based on a sample of 2483 individuals. Two missing in the variable socioeconomic position

**Fig. 1:**
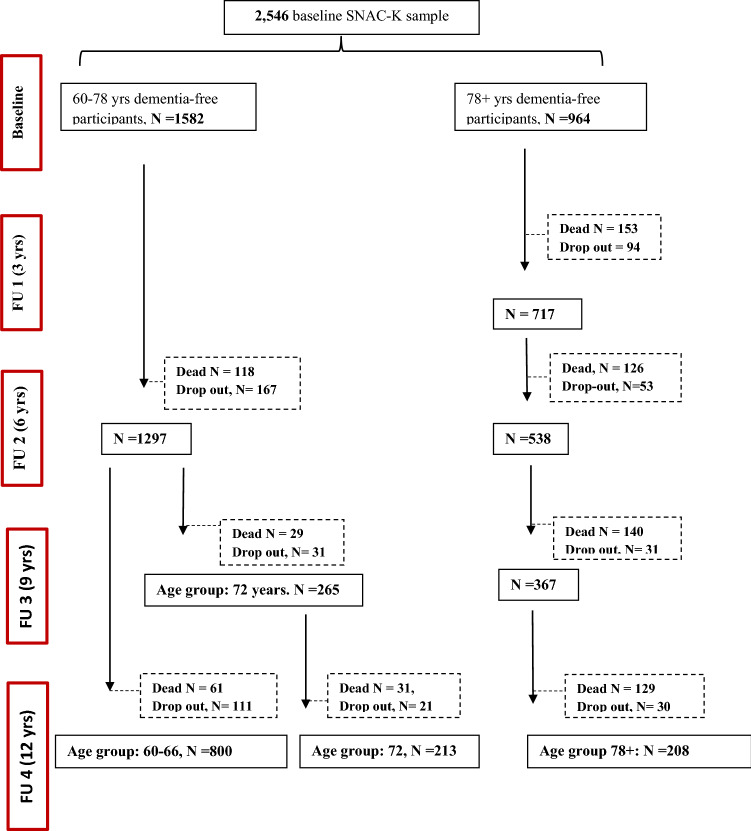
Flowchart of study participation over 12 years. Dropouts are due to either refusal of the participant/relative, loss of contact with the participant, or moving of the participant from the city where the study took place

By the end of the follow-up (mean: 11.5 ± 4.4 years), 787 people had died (239 among the young cohort and 548 among the older one) and 538 had dropped out (330 among those in the younger cohort and 208 among those 78+). Figure 1 depicts the flowchart of the study participation.

**Fig. 2:**
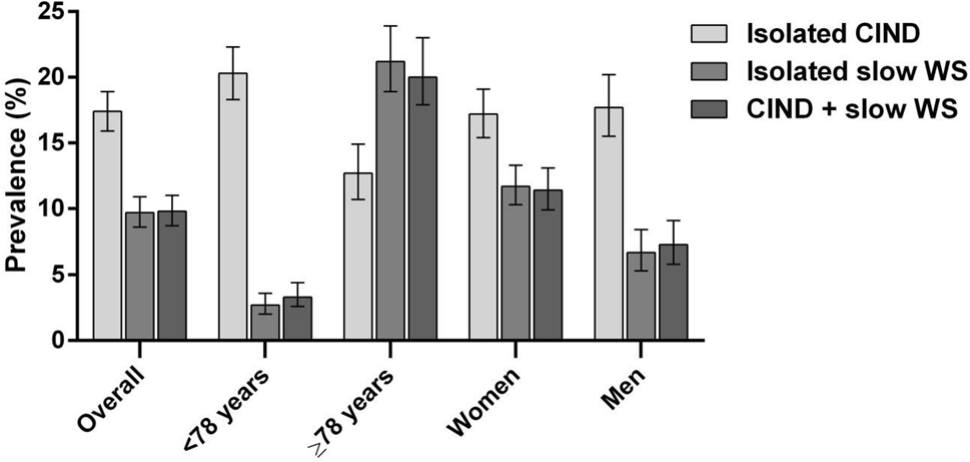
Prevalence per 100 participants of functional profiles at baseline in the Swedish National study on Aging and Care-Kungsholmen (SNAC-K), Stockholm, Sweden. CIND, Cognitive impairment, no dementia; WS, walking speed

Figure 2 shows the distribution of functional profiles by age group and sex. Of the participants, 1613 (64%) presented a healthy functional profile, 441 (17%) had isolated CIND, 255 (10%) had isolated slow WS, and 237 (9%) had both conditions.

Participants with both CIND and slow WS were older (*p* = .049), more likely to be women (*p* < .01), and more likely to have a lower level of education (*p* < .01) than those presenting a healthy functional profile. We did not observe any statistically significant differences in the number of diseases across the different groups (*p* = .707).

**Fig. 3:**
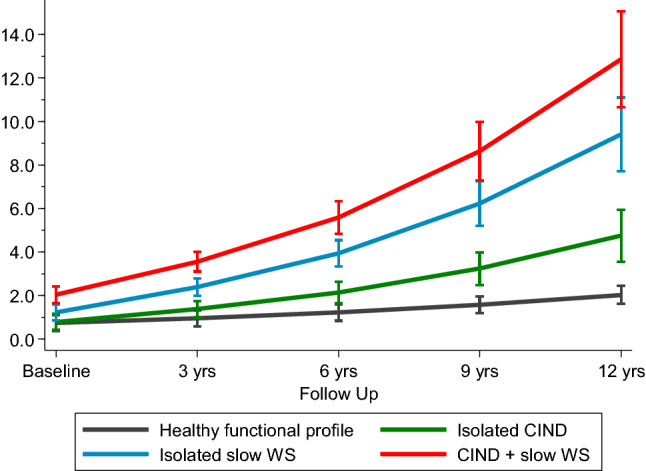
Trajectories with 95% confidence intervals of disability over 12 years of follow-up by functional profiles. CIND, Cognitive impairment, no dementia; slow WS: walking speed < 0.8 m/s. Trajectories were derived from multilevel mixed-effect linear regression models adjusted for age, sex, education, socioeconomic position, physical inactivity, time to death, cardio- and cerebrovascular diseases, hypertension, depression and mood disorders, solid neoplasms, chronic obstructive pulmonary diseases, and malnutrition. Healthy functional profile is intended as participants without CIND and with a walking speed ≥ 0.8 m/s

Figure 3 depicts the trajectories of disability over time, controlling for age, sex, education, time to death, chronic diseases, and malnutrition. Participants with both CIND and slow WS had the steepest increase in the disability score (*β*: 0.46, *p* < .001), followed by people with isolated slow WS (*β*: 0.36, *p* < .001) and people with isolated CIND (*β*: 0.08, *p* < .001).

Adjusted HRs of mortality by functional profile are reported in Table 2. Participants with both CIND and slow WS had the highest mortality rate, and this association was stronger in the short-term follow-up (≤ 3 years). Within 3 years of follow-up, the mortality rate in people with deficits in both domains was five times higher (HR 5.1; 95% CI 3.5–7.4) than that of participants with a healthy functional profile. Between 3 and 6 years of follow-up, it was four times higher (HR 4.1; 95% CI 3.0–5.6). The association between the co-occurrence of CIND and slow WS and mortality was attenuated in the long term. The association between isolated CIND and isolated slow WS and survival remained relatively stable over follow-up; results were slightly mitigated over the long term.

**Table 2:** Hazard ratios of mortality with 95% confidence intervals by functional profiles

Functional profiles	Mortality					
	0–3 years of follow-up		3–6 years of follow-up		6+ years of follow-up	
	Events/at risk	HR (95% CI)	Events/at risk	HR (95% CI)	Events/at risk	HR (95% CI)
Healthy functional profile^a^	49/1613	1.00 (Ref)	99/1564	1.00 (Ref)	389/1465	1.00 (Ref)
Isolated CIND	25/441	1.8 (1.1–2.9)	41/416	1.6 (1.1–2.2)	135/375	1.6 (1.3–2.0)
Isolated slow WS	39/255	2.4 (1.6–3.6)	54/216	2.0 (1.4–2.8)	112/162	1.6 (1.3–2.0)
CIND+ slow WS	68/237	5.1 (3.5–7.4)	71/169	4.1 (3.0–5.6)	75/98	2.4 (1.9–3.1)

Models adjusted for age, sex, education, socioeconomic position, physical inactivity, cardio- and cerebrovascular diseases, hypertension, depression and mood disorders, solid neoplasms, chronic obstructive pulmonary diseases, and malnutrition

*HR* hazard ratio, *CI* confidence interval, *CIND* cognitive impairment, no dementia, *WS* walking speed

^a^Participants without CIND and with a walking speed ≥ 0.8 m/s

When we estimated the PAR for each of the functional profiles we found that, within 3 years of follow-up, 31.3% (95% CI 28.4–34.2%) of deaths could be attributed to co-occurring CIND and slow WS at baseline, 6.4% (95% CI 2.3–10.3%) of death could be related to the presence of isolated CIND, and 13.3% (95% CI 9.0–17.5%) to isolated slow WS. Overall, 45.7% (95% CI 33.4–55.7%) of death could be attributed to the presence of at least one of the functional profiles at baseline.

*Sensitivity analyses* Consistent results have been obtained when the functional profiles were considered as time-changing variables over time. We found a higher disability score in people with co-occurring CIND and slow WS, followed by people with isolated slow WS and a non-statistically significant higher disability score in people with isolated CIND (for both CIND and slow WS: *β*: 1.79; 95% CI 1.66; 1.91; for isolated slow WS: *β*: 0.76; 95% CI 0.66; 0.87; for isolated CIND; *β*: 0.08; 95% CI − 0.003; 0.17). Similarly, the HRs for mortality considering the functional profiles as time-changing variables over time, we found that people with both CIND and slow WS had the highest HR (3.56; 95% CI 3.00–4.21), followed by isolated slow WS (HR 1.94; 95% CI 1.63; 2.29) and isolated CIND (HR 1.88; 95% CI 1.59–2.23).

Figure S2 shows the trajectories of disability over time for the different functional profiles, excluding people with incident dementia within the first 6 years of follow-up. Consistent results, although attenuated, have been obtained.

The magnitude and the direction of the estimates based on complete cases and multiple imputation were similar overall both for time to death and disability (data not shown).

For disability, the multi-adjusted joint models resulted in stronger associations with the same direction as the main results (Table S1). In particular, participants with both CIND and slow WS had the steepest increase in disability score with an estimate of the *β* coefficient of 1.16 (*p* < .001). The point estimate of the *β* coefficient was also slightly stronger for isolated CIND (*β*: 0.11 in the joint model). Conversely, *β* coefficients for isolated slow WS were attenuated in the joint models.

Table S2 shows the *β* coefficients at baseline and over the 12 years of follow-up when we considered as outcome the changes in ADL and IADL scores separately. Participants with both CIND and slow WS experienced the steepest accumulation of impaired ADL or IADL over time (*p* < .001 for all).

When we repeated the analyses with moderate to severe CIND and a different cutoff for slow WS (i.e., 1 m/s), the results were consistent (data not shown). In the stratified analyses, the association between the exposure and both of the outcomes of interest was stronger in the older group. Conversely, no differences were detected when we stratified by sex (Tables S3 and S4).

## Discussion

The results of the present study support our hypothesis that older adults who present simultaneously with cognitive and physical impairments are at higher risk for negative health-related events. People with this functional profile had the steepest disability development and the highest mortality rate. Up to half of all deaths that occurred during the first 3 years of follow-up could be related to the presence of one of the functional profiles investigated in the study (i.e., either isolated CIND, isolated slow WS or both CIND and slow WS). The combination of CIND and slow WS accounted for almost two-thirds of these deaths. In general, results were attenuated over the longer follow-up.

Taken as a whole, our findings add weight to the existing evidence that functional deficits are good predictors of disability and mortality, pointing out the need for a combined assessment of cognitive and physical function to capture deterioration in health. In addition, for the first time we here report the prognosis of cognitive and physical deficits over short- and long-term follow-up, and trajectories of functional decline. Notably, the combination of CIND and slow WS conferred a greater accumulation of disability and a higher mortality rate also with respect to having isolated CIND and—with a marginal statistical significance—to having isolated slow WS. Both clinical and public health implications stem from these results. Clinicians might better stratify the short- and long-term risk for further deterioration in health of the older patient with cognitive and physical problems. From a public health standpoint, these findings might provide valuable information for better planning and implementing individualized preventive and therapeutic strategies.

Aging is a complex phenomenon characterized by a set of multifaceted—sometimes subclinical—biological dysfunctions that may have an impact on both cognitive and physical functioning [4, 37]. As a consequence, measures of cognition or physical function alone seem inadequate to capture the large heterogeneity of health and its changes in the older population. A number of longitudinal studies have investigated the simultaneous impact of cognitive and physical impairment on negative outcomes in older adults [6, 7, 9, 38]; their results have been inconsistent. Notably, two previous population-based studies that compared robust older adults to those with co-occurring cognitive and physical impairments did not observe an association between such impairments and dependence or shorter survival [10, 11]. The authors of one of those studies suggested that the short follow-up (4 years) may have prevented them from demonstrating that co-occurring cognitive impairment and physical frailty were negatively associated with mortality. Our results do not seem to support this interpretation, since we found the highest mortality rate in the first 3 years of observation. Other groups [6, 9, 39] have reported a higher risk of adverse outcomes, including poor quality of life, dementia, disability and mortality, in older adults with low MMSE scores and physical frailty, compared with robust ones. Our results are also in line with the findings of the Gait and Brain study, the Italian Longitudinal Study on Aging (ILSA), and the Singapore Study on Aging [12, 38, 40]. Those studies suggest that the predictive validity with regard to adverse health outcomes is better when cognition and physical function are considered simultaneously. It is plausible that the steeper accumulation of disabilities that we observe in people with CIND (with or without slow WS) might be mediated by the longitudinal development of dementia, which would be in line with evidence showing faster development of dementia in people with initial cognitive impairment, especially when associated with slow gait speed [41].

Often, in population-based settings, cognitive impairment is assessed through measures of global cognitive function (e.g., MMSE, MoCA) or through the presence of subjective cognitive complaints [42]. These measures are easily administered, inexpensive, and reliably identify cognitive impairment in the general population. However, a detailed neuropsychological battery is more sensitive to mild and initial cognitive deficits, making it possible to identify people with worse clinical prognosis. Additionally, frail older adults can have specific impairments in memory, attention and executive function, and neuropsychological assessment provides more detailed information on these cognitive domains [43].

In this study, we chose WS to assess physical function. As shown by Santoni et al. [44], slow WS properly discriminates older adults’ health across different ages. The ability to walk is predicated on the integrity and coordination of several systems, in particular the nervous, cardio-respiratory and musculoskeletal systems [13]. Slow WS has been associated with poor cognitive performance and higher dementia incidence in several studies [45]. Moreover, WS is a good to excellent overall predictor of survival (area under the curve ranging from 0.66 to 0.82) [13, 14]. Although the relationship between WS and survival seems continuous, the use of a cutoff point may help interpretation. Several authors have proposed that a WS faster than 1.0 m/s suggests better than average life expectancy, whereas speeds slower than 0.6 m/s greatly increase the likelihood of poor health and function. Studenski et al. [13] suggest that an intermediate cutoff of 0.8 m/s could be used to identify an at-risk subgroup of people with shorter life expectancy. Since WS can be assessed by non-professionals using only a walkway and a stopwatch, it is also simpler to conduct than many other clinical assessments.

Studies have explored a number of biological mechanisms that may explain the link between impairments in cognition and physical function [46–48]. Executive cognitive and motor functions rely on common brain regions and networks: slower WS has been associated with smaller prefrontal regions and basal ganglia, and balance difficulty might be related to dysfunction in the cerebellum [49]. A possible explanation of the co-occurrence of cognitive and physical impairments might stem from the damages in common brain regions, and physical and cognitive decline can be seen as downstream consequences of such dysfunctions [48]. Understanding gait and cognitive impairments as a result of the underlying processes affecting function in common brain regions might help in the identification of those modifiable factors including vascular damage, chronic inflammation, and neurodegeneration. At the same time, the co-occurrence of physical and cognitive dysfunction might be the results of a greater disease burden, or the presence of a single—but severe—disease. As a consequence, the accumulation of diseases, the presence of systemic inflammation or cellular dysfunctions might accelerate the decline in physical and cognitive capacities resulting—in turn—in an increased disability and a shorter survival.

Despite the underlying putative biological link between cognition and physical function, growing evidence consistently points toward the clinical relevance of conducting multidimensional health assessments in older adults.

Major strengths of our study include the large population-based sample, clinical assessment by physicians and nurses, extensive neuropsychological battery to assess cognitive performance, repeated measures of the exposures and the outcomes, and a 12-year follow-up. However, some limitations need to be mentioned. Those who dropped out during the study period were more likely to survive for a shorter period of time and to be older, have more complex health status, and be frail than those who participated throughout the entire follow-up period. To take such informative dropouts into account, we jointly modeled the longitudinal and survival outcomes. The direction of the effects was similar, and as expected the point estimates were even stronger, supporting the possibility of an underestimation of the association when death is not taken into account as competing event of the analyses. We also repeated the analyses using multiple imputations for missing baseline data. The estimates derived from complete cases and multiple imputations suggest that missing data had little role in the observed findings. Third, although we adjusted the analyses for major confounders, we cannot completely rule out the presence of residual confounding. Finally, SNAC-K includes older adults in central Stockholm who are of high socioeconomic status and are fit and healthy, which might limit the generalizability of our results to other populations.

In conclusion, the results of this study suggest that clinically assessing cognitive and physical function may ease the identification of people at higher risk for adverse events. Our findings have several clinical and public health implications, and point toward the need to further explore the risk factors, pathways, and biological mechanisms underlying the co-occurrence of mental and physical dysfunction. Such research might help identify a segment of the population that deserves specific assessment and care, and enable the development of personalized intervention programs and preventive strategies.

## Electronic supplementary material

Below is the link to the electronic supplementary material.

Supplementary material 1 (DOCX 116 kb)
