# Supplementary material for: Disability trajectories and mortality in older adults with different cognitive and physical profiles
Source: Aging Clin Exp Res. 2019 Aug 30;32(6):1007–16. doi: 10.1007/s40520-019-01297-1 (PMC7260142; doi:10.1007/s40520-019-01297-1)
Supplement: Supplementary file 1 — Supplementary material 1 (DOCX 116 kb) [file 40520_2019_1297_MOESM1_ESM.docx]

**Supplementary files**

**Figure S1.** Flowchart of the study population in the Swedish National Study on Aging and Care–Kungsholmen (SNAC-K), Stockholm, Sweden

**Table S1**. Joint models. Outcome: disability.

**Table S2**. β-coefficients and 95% confidence intervals (95% CI) for the associations of different functional profiles with baseline (intercept) activities of daily living (ADL) and instrumental activities of daily living (IADL) and annual changes (slope) over 12 years, from multivariable mixed-effect models.

**Table S3**. Main analysis stratified by age and sex. Outcome: disability.

**Table S4**. Main analysis stratified by age and sex. Outcome: death.

**Figure S2. Trajectories with 95% confidence intervals of disability over 12 years of follow-up by functional profiles, after excluding incident cases of dementia within the first six years**

**Figure S1** Flowchart of the study population in the Swedish National Study on Aging and Care–Kungsholmen, Stockholm, Sweden

**Study participants**

**3363** participants aged ≥60 years

**2848** participants underwent the neuropsychological assessment

390 declined the neuropsychological assessment

10 died before the neuropsychological assessment

106 with MMSE scores of <10

9 did not complete the neuropsychological assessment because of severe vision or earing problems

**Baseline survey**

2001−2004

129 missing data in one or more cognitive domain

77 missing data on walking speed

**2546 final sample**

 83 prevalent dementia cases

11 participants with schizophrenia

2 participants with intellectual disability/neurodevelopmental disorder

**2752** dementia-free participants

**Table S1.** Differences in disability score with 95% confidence intervals derived from mixed models and from joint model by functional profiles.

|  | **Disability** | |
| --- | --- | --- |
|  | β (95% CI)  Mixed-effect model | β (95% CI)  Joint model |
| **Functional profiles (baseline)** |  |  |
| Healthy functional profile* | Ref. | Ref. |
| Isolated CIND | 0.14 (0.02; 0.26) | 0.11 (-0.32; 0.54)^ns^ |
| Isolated slow WS | 0.52 (0.36; 0.67) | 0.52 (0.13; 0.92) |
| CIND+ slow WS | 1.82 (1.65; 2.20) | 1.83 (1.36; 2.29) |
| **Functional profiles** **(Δ disability decline x time )** | |  |
| Healthy functional profile* | Ref. | Ref. |
| Isolated CIND | 0.08 (0.04; 0.12) | 0.11 (0.04; 0.18) |
| Isolated slow WS | 0.37 (0.31; 0.43) | 0.14 (0.07; 0.22) |
| CIND+ slow WS | 0.46 (0.38; 0.54) | 1.16 (0.07-1.25) |
|  |  |  |

*β-coefficients were derived from multilevel mixed-effect linear regression models adjusted for age, sex, education,* ***socio-economic position, physical inactivity****, time to death, cardio- and cerebrovascular diseases, hypertension, chronic obstructive pulmonary disorders, solid neoplasms,* ***depression*** *and mood disorders, and malnutrition.*

CIND, cognitive impairment, no dementia; WS, walking speed; CI, confidence interval; ns, not significant

* Participants without CIND and with a WS ≥0.8 m/s.

**Table S2** β-coefficients and 95% confidence intervals (95% CI) for the associations of different functional profiles with baseline (intercept) activities of daily living (ADL) and instrumental activities of daily living (IADL) and annual changes (slope) over 12 years, from multivariable mixed-effect models.

|  | IADL impaired | ADL impaired |
| --- | --- | --- |
|  | β (95% CI) | β (95% CI) |
| **Functional profiles (at baseline)** |  |  |
| Healthy functional profile* | Ref. | Ref. |
| Isolated CIND | 0.14 (0.03; 0.24) | 0.02 (-0.03; 0.06)^ns^ |
| Isolated slow WS | 0.52 (0.38; 0.65) | -0.03 (-0.08; 0.03) ^ns^ |
| CIND+ slow WS | 1.67 (1.51; 1.81) | 0.13 (0.07; 0.19) |
| **Functional profiles (Δ disability decline x time)** | |  |
| Healthy functional profile* | Ref. | Ref. |
| Isolated CIND | 0.05 (0.03; 0.08) | 0.03 (0.01; 0.04) |
| Isolated slow WS | 0.25 (0.20; 0.29) | 0.13 (0.10; 0.15) |
| CIND+ slow WS | 0.28 (0.22; 0.32) | 0.20 (0.17; 0.22) |
|  |  |  |

Abbreviations: IADL: instrumental activities of daily living; ADL: basic activities of daily living; CIND: cognitive impairment no dementia; WS: walking speed

ns: not statistically significant

*β-coefficients were derived from multilevel mixed-effect linear regression models adjusted for age, sex, education,* ***socio-economic position, physical inactivity****, time to death, cardio- and cerebrovascular diseases, hypertension, chronic obstructive pulmonary disorders, solid neoplasms,* ***depression*** *and mood disorders, and malnutrition.*

* Participants without CIND and with a WS ≥0.8 m/s.

**Table S3.** Differences in disability score with 95% confidence intervals derived by fully adjusted mixed models by functional profiles, stratified by sex (A) and age (B).

(A) Analysis stratified by sex

|  | **Baseline disability (95% CI)** | **Baseline disability (95% CI)** | **Δ disability x time (95% CI)** | **Δ disability x time (95% CI)** |
| --- | --- | --- | --- | --- |
|  | **Females** | **Males** | **Females** | **Males** |
| **Healthy functional profile*** | Ref | Ref | Ref | Ref |
| **Isolated CIND** | 0.11 (-0.05; 0.27) | 0.17 (-0.00; 0.35) | 0.08 (0.02; 0.13) | 0.04 (-0.03; 0.11) |
| **Isolated slow WS** | 0.32 (0.12; 0.52) | 0.51 (0.24; 0.80) | 0.34 (0.27; 0.42) | 0.29 (0.17; 0.41) |
| **CIND + slow WS** | 1.70 (1.47; 1.91) | 1.41 (1.13-1.69) | 0.55 (0.46; 0.64) | 0.50 (0.36; 0.63) |

p for interaction between functional profiles and sex: not statistically significant

(B) Analysis stratified by age

|  | **Baseline disability (95% CI)** | | **Baseline disability (95% CI)** | | **Δ disability x time (95% CI)** | | **Δ disability x time (95% CI)** | |  |
| --- | --- | --- | --- | --- | --- | --- | --- | --- | --- |
|  | <78 years | | 78+ | | <78 years | | 78+ | |  |
| **Healthy functional profile*** | | Ref | | Ref | | Ref | | Ref | |
| **Isolated CIND** | | 0.04 (-0.04; 0.12) | | 0.35 (0.04; 0.65) | | 0.04 (0.01; 0.07) | | 0.21 (0.07; 0.33) | |
| **Isolated slow WS** | | 0.21 (0.01; 0.42) | | 0.74 (0.48; 1.00) | | 0.11 (0.03; 0.18) | | 0.23 (0.12; 0.34) | |
| **CIND + slow WS** | | 0.46 (0.27; 0.65) | | 2.31 (2.03; 2.59) | | 0.45 (0.37; 0.53) | | 0.45 (0.32; 0.59) | |

p for interaction between functional profiles and age: <0.05

Models adjusted for age, sex, education, **socio-economic position**, **physical inactivity**, cardio- and cerebrovascular diseases, hypertension, chronic obstructive pulmonary disorders, solid neoplasms, **depression** and mood disorders, and malnutrition. CIND, cognitive impairment, no dementia; WS, walking speed; CI, confidence interval; ns, not significant.

*Participants without CIND and with a WS ≥0.8 m/s.

**Table S4** Hazard ratios of mortality with 95% confidence intervals by functional profiles, stratified by sex (A) and age (B).

(A) Analysis stratified by sex

|  | **HR (95% CI) of mortality** | | | | | |
| --- | --- | --- | --- | --- | --- | --- |
|  | **0-3 years** | | **3-6 years** | | **6+ years** | |
|  | **Females** | **Males** | **Females** | **Males** | **Females** | **Males** |
| **Healthy functional profile*** | Ref | Ref | Ref | Ref | Ref | Ref |
| **Isolated CIND** | 1.2 (0.6-2.5) | 2.3 (1.2-4.3) | 1.3 (0.8-2.2) | 1.5 (0.9-2.5) | 1.2 (0.9-1.6) | 1.5 (1.-2.0) |
| **Isolated slow WS** | 1.5 (0.8-2.8) | 3.0 (1.6-5.9) | 1.8 (1.2-2.8) | 2.1 (1.2-3.7) | 1.3 (1.0-1.7) | 2.0 (1.3-3.1) |
| **CIND + slow WS** | 3.8 (2.3-6.3) | 3.3 (1.8-6.1) | 3.3 (2.2-4.9) | 2.4 (1.4-4.3) | 1.9 (1.4-2.6) | 1.8 (1.1-2.8) |

p for interaction between functional profiles and sex: not statistically significant

(B) Analysis stratified by age

|  | **HR (95% CI) of mortality** | | | | | |
| --- | --- | --- | --- | --- | --- | --- |
|  | **0-3 years** | | **3-6 years** | | **6+ years** | |
|  | **<78 years** | **78+** | **<78 years** | **78+** | **<78 years** | **78+** |
| **Healthy functional profile*** | Ref | Ref | Ref | Ref | Ref | Ref |
| **Isolated CIND** | 2.3 (1.1-4.8) | 1.9 (1.0-3.6) | 1.7 (1.0-2.9) | 1.6 (0.9-2.6) | 1.5 (1.2-2.1) | 1.8 (1.4-2.4) |
| **Isolated slow WS** | 1.2 (0.2-8.7) | 2.6 (1.6-4.3) | 2.9 (1.2-6.9) | 2.3 (1.5-3.5) | 1.7 (0.9-3.3) | 1.5 (1.2-1.9) |
| **CIND + slow WS** | 4.4 (1.6-12.0) | 5.9 (3.8-9.2) | 4.0 (2.0-8.1) | 4.8 (3.3-6.9) | 2.0 (1.5-2.7) | 4.0 (2.6-6.5) |

p for interaction between functional profiles and age: <0.05.

Models adjusted for age, sex, education, **socio-economic position**, **physical inactivity**, cardio- and cerebrovascular diseases, hypertension, chronic obstructive pulmonary disorders, solid neoplasms, **depression** and mood disorders, and malnutrition. CIND, cognitive impairment, no dementia; WS, walking speed; CI, confidence interval. * Participants without CIND and with a walking speed ≥0.8 m/s

**Figure S2. Trajectories with 95% confidence intervals of disability over 12 years of follow-up by functional profiles, after excluding incident cases of dementia within the first six years**

***CIND, Cognitive impairment, no dementia; slow WS: walking speed <0.8 m/s.***

***Trajectories were derived from multilevel mixed-effect linear regression models adjusted for age, sex, education, socio-economic position, physical inactivity, time to death, cardio-and cerebrovascular diseases, hypertension, depression and mood disorders, solid neoplasms, chronic obstructive pulmonary diseases, and malnutrition.***

***Healthy functional profile is intended as participants without CIND and with a walking speed ≥0.8 m/s.***
